# Global citrus root microbiota unravels assembly cues and core members

**DOI:** 10.3389/fmicb.2024.1405751

**Published:** 2024-07-26

**Authors:** Monia F. Lombardo, Yunzeng Zhang, Jin Xu, Pankaj Trivedi, Pengfan Zhang, Nadia Riera, Lei Li, Yayu Wang, Xin Liu, Guangyi Fan, Jiliang Tang, Helvécio D. Coletta-Filho, Jaime Cubero, Xiaoling Deng, Veronica Ancona, Zhanjun Lu, Balian Zhong, M. Caroline Roper, Nieves Capote, Vittoria Catara, Gerhard Pietersen, Abdullah M. Al-Sadi, Xun Xu, Jian Wang, Huanming Yang, Tao Jin, Gabriella Cirvilleri, Nian Wang

**Affiliations:** ^1^Citrus Research and Education Center, Department of Microbiology and Cell Science, Institute of Food and Agricultural Sciences (IFAS), University of Florida, Lake Alfred, FL, United States; ^2^Department of Agriculture, Food and Environment (Di3A), University of Catania, Catania, Italy; ^3^College of Bioscience and Biotechnology, Yangzhou University, Yangzhou, Jiangsu, China; ^4^Department of Bioagricultural Sciences and Pest Management, Colorado State University, Fort Collins, CO, United States; ^5^BGI-Shenzhen, Shenzhen, Guangdong, China; ^6^The State Key Laboratory for Conservation and Utilization of Subtropical Agro-Bioresources, College of Life Science and Technology, Guangxi Key Laboratory for Sugarcane Biology, Guangxi University, Nanning, Guangxi, China; ^7^Instituto Agronômico, IAC Centro de Citricultura Sylvio Moreira, CCSM, Cordeirópolis, São Paulo, Brazil; ^8^Department of Plant Protection, National Institute for Agricultural and Food Research and Technology (INIA-CSIC), Madrid, Spain; ^9^Department of Plant Pathology, South China Agricultural University, Guangzhou, China; ^10^Texas A&M University-Kingsville Citrus Center, Weslaco, TX, United States; ^11^National Navel Orange Engineering Research Center, Gannan Normal University, Ganzhou, Jiangxi, China; ^12^Department of Microbiology and Plant Pathology, University of California, Riverside, Riverside, CA, United States; ^13^IFAPA Las Torres, Seville, Spain; ^14^Department of Genetics, University of Stellenbosch, Stellenbosch, South Africa; ^15^Department of Plant Sciences, Sultan Qaboos University, Muscat, Oman

**Keywords:** endophytes, beneficial microorganisms, core microbiome, rootstock selection, biological control agents

## Abstract

**Introduction:**

Citrus is one of the most important fruit crops worldwide, and the root-associated microbiota can have a profound impact on tree health and growth.

**Methods:**

In a collaborative effort, the International Citrus Microbiome Consortium investigated the global citrus root microbiota with samples collected from nine citrus-producing countries across six continents. We analyzed 16S rDNA and ITS2 amplicon sequencing data to identify predominant prokaryotic and fungal taxa in citrus root samples. Comparative analyses were conducted between root-associated microbial communities and those from the corresponding rhizosphere and bulk soil samples. Additionally, genotype-based group-wise comparisons were performed to assess the impact of citrus genotype on root microbiota composition.

**Results:**

Ten predominant prokaryotic phyla, containing nine bacterial phyla including Proteobacteria, Actinobacteria, Acidobacteria, and Bacteroidetes and one archaeal phylum (Thaumarchaeota), and multiple fungal phyla including Ascomycota and Basidiomycota were identified in the citrus root samples. Compared with the microbial communities from the corresponding rhizosphere and bulk soil samples from the same trees, the prokaryotic and fungal communities in the roots exhibited lower diversity and complexity but greater modularity compared to those in the rhizosphere. In total, 30 root-enriched and 150 root-depleted genera in bacterial community were identified, whereas 21 fungal genera were enriched, and 147 fungal genera were depleted in the root niche compared with the rhizosphere. The citrus genotype significantly affected the root prokaryotic and fungal communities. In addition, we have identified the core root prokaryotic genera comprising *Acidibacter, Allorhizobium, Bradyrhizobium, Chitinophaga, Cupriavidus, Devosia, Dongia, Niastella, Pseudomonas, Sphingobium, Steroidobacter* and *Streptomyces*, and the core fungal genera including *Acrocalymma, Cladosporium, Fusarium, Gibberella, Mortierella, Neocosmospora* and *Volutella*. The potential functions of these core genera of root microbiota were predicted.

**Conclusion:**

Overall, this study provides new insights into the assembly of microbial communities and identifies core members of citrus root microbiota across a wide geographic range. The findings offer valuable information for manipulating root microbiota to enhance plant growth and health.

## Introduction

A vast number of microbes inhabit the root-associated niches, including the rhizosphere, rhizoplane, and endosphere, collectively known as root-associated microbiomes (Trivedi et al., 2020). These microbes can be categorized to be mutualists, pathogens, or commensals, depending on how they interact with the plant hosts (Thoms et al., 2021). While pathogens can lead to specific diseases after colonization and commensals are usually loosely dependent on the hosts, mutualists can benefit plant hosts through several means, such as helping plants absorb mineral nutrients, resisting against pathogen invasion, and promoting abiotic and biotic stress tolerance, and thus gain increasing attention in the field of agriculture research (Vandenkoornhuyse et al., 2015; Afridi et al., 2022). A few mutualistic microbes have been identified in the root-associated microbiomes. For instance, several fungal members have been observed to establish arbuscular mycorrhizal (AM) symbiosis, aiding in the absorption of phosphate by plants (Bennett and Groten, 2022); specific bacterial members such as rhizobia possess the ability to perform biological nitrogen fixation and provide nitrogen nutrients to plants (Pankievicz et al., 2019).

Identification and application of mutualistic microbes is an effective strategy for more productive and sustainable agriculture (Mueller and Sachs, 2015). Inoculation of beneficial microbe has been widely performed in recent few decades; however, root-associated niches are recognized as highly competitive habitats, which may result in inefficient colonization of the inoculated microbes, and inefficiency of the inoculated microbes is indeed frequently observed in field trails (Timofeeva et al., 2023; Wang et al., 2023). The competitive stresses in the root-associated niches mainly result from the plant-derived selective pressures and the fierce inter-microbe competitions (Reinhold-Hurek et al., 2015; Hassani et al., 2018; Dimaria et al., 2023). Therefore, plant genotype and the soil properties have significant effects on the root microbiota composition and the efficiency of inoculated microbes (French et al., 2020). Under such circumstances, understanding the assembly cues of the root-associated microbiota and identification of beneficial microbes in the “core” microbiota (i.e., a subset of the plant microbiota that is reproducibly associated with a particular crop host across different genotypes and soil properties) is a promising approach for development of beneficial microbe inoculation-based agricultural practices (Risely, 2020; Neu et al., 2021; Mapelli et al., 2023). In fact, the “core” members usually exhibit a greater adaptability and persistence to the host’s environments and form stronger mutual relationship with the host, compared to less stable or less host-specific microorganisms in the microbiota (Lemanceau et al., 2017). The elucidation of core members and their functions have already provided a strong basis for developing effective probiotics or for manipulation of microbial communities for agricultural benefits (Blaustein et al., 2017; Trivedi et al., 2020; Zhang et al., 2021; Park et al., 2023). Some preliminary laboratory-based results have yielded encouraging results regarding the applications of selected core members for their antagonistic activity against plant pathogens or plant growth promotion (Tian et al., 2017; Munir et al., 2020; Jing et al., 2023). Importantly, how to best utilize plant microbiomes has been deemed to be one of the key challenges by the global plant pathology community (Wang et al., 2024).

Citrus (*Citrus* spp.) is one of the most economically significant fruit crops worldwide, and the root-associated microbiota can have a profound impact on tree health and fruit production (Riera et al., 2017; Bai et al., 2019; Zhang et al., 2021; Jing et al., 2023). Numerous studies investigated the citrus microbiome composition of the belowground niche (Xu et al., 2018, 2023; Wu et al., 2020; Xi et al., 2023), especially in the context of Huanglongbing (HLB) disease (Trivedi et al., 2010, 2012; Blaustein et al., 2017; Zhang et al., 2017; Ginnan et al., 2018). Moreover, core members and their functions primarily associated with the rhizosphere were extensively described (Blaustein et al., 2017; Xu et al., 2018; Ginnan et al., 2020; Wu et al., 2020). Several microbes in the root microbiota have been demonstrated to promote plant fitness by producing plant growth-promoting substances, such as auxins, cytokinins, and gibberellins. They can also protect plants from biotic and abiotic stresses, such as drought, salinity, and pathogen attacks by producing secondary metabolites (Eljounaidi et al., 2016; Tian et al., 2017; Carrión et al., 2019; Khan et al., 2019). However, compared to the rhizosphere, the core members of root microbiota of citrus, which form more intimate interactions with the plant host, remain relatively unexplored. Revealing of the assembly cues and identification of the core members and functions of the citrus root microbiota at a global scale are crucial in steering research toward development of beneficial microbe inoculation-based approaches to citrus production industry such as disease management.

In this study, we delved into the citrus root-associated microbiota on a global scale with the aims to (i) unravel the microbial composition and diversity of citrus root microbiota; (ii) investigate the role of host genotypes in influencing the root microbiota; and (iii) characterize the core members and their associated functions. Our results provide valuable insights to understand the assembly cues of citrus root microbiota and facilitate the identification of beneficial microbes for manipulating the citrus microbiota to enhance plant growth, stress tolerance and disease management.

## Materials and methods

### Experimental design, sample collection, and amplicon sequencing

Citrus root samples were collected from 28 distinct citrus groves situated across nine citrus-producing countries (Supplementary Table S1), encompassing all six continents that citrus can grow. The root samples were collected from local citrus varieties (scion/rootstock) in each location (Supplementary Table S1). For each country, the most representative citrus growing locations were selected for samples collection, except for Oman, which had five locations. In each grove, four healthy trees were selected and, from each of these trees, fine roots (approximately 1 mm diameter) from a depth of 5–15 cm were collected. For each tree, the samples were collected from four ordinate directions approximately 1 meter away from the trunk. The roots were removed from the soil with a shovel and then gently shaken to remove the soil that was not tightly attached to the roots. The roots from the four locations/tree were pooled and washed thrice using PBS buffer. 2 grams of washed roots were subjected for DNA extraction using a MoBio Powersoil DNA extraction kit (MoBio Laboratories Inc. Carlsbad, CA, USA) according to the manufacturer’s instructions. The DNA quality and quantity were tested using a NanoDrop device (Thermo Scientific, Wilmington, DE, USA) and through electrophoresis (0.8% agarose gel, including a 1 kb plus ladder). The DNA samples from the four trees collected from the same grove were pooled together and stored at −80°C until use. It is noteworthy that the root samples (including rhizoplane and endosphere) were collected with the rhizosphere (soil surrounding the roots), and bulk soil (Supplementary Table S1), which allows comparison with rhizosphere and bulk soil samples from same locations. Analyses of the bulk soil and rhizosphere samples were already reported in our previous study (Xu et al., 2018).

To investigate both prokaryotic and eukaryotic microbes in the root samples, 16S and ITS2 amplicon library preparation and sequencing were performed according to the manufacturer’s protocol at BGI-Shenzhen. For targeted metabarcoding sequencing, DNA fragments were amplified using primers targeting the 16S rDNA V4 region (515F and 806R) (Caporaso et al., 2011) and fungal ITS2 (Tedersoo et al., 2015). After quality control, quantification, and normalization of the DNA libraries, 250 bp paired-end reads were generated using the Illumina MiSeq250 sequencing platform according to the manufacturer’s instructions. The raw sequencing reads of root samples were deposited in the NCBI Short Read Archive BioProject PRJNA844917. For the comparative analysis with rhizosphere and soil, raw sequencing reads of the corresponding rhizosphere and bulk soil samples were obtained from Xu et al. (2018) (BioProject PRJNA362455).

### Amplicon data analysis

The 16S rRNA gene and ITS2 amplicon sequencing data of the root samples were analyzed using the DADA2 pipeline (R package dada2, v.1.8.0) (Callahan et al., 2016) in R software (v.4.2) as follows. To compare with corresponding rhizosphere and bulk soil samples from previous study (Xu et al., 2018), we also included the bacterial 16S rDNA and fungal ITS2 amplicon data of rhizosphere and bulk soil samples for further analyses using the same methods as root samples. In detail, the raw sequence reads were first filtered, de-replicated and de-noised using DADA2 recommended parameters. Paired-end sequences were then merged, and chimeras were removed. To obtain the taxonomic information, representative sequences of ASVs were searched against SILVA database for 16S and UNITE database for ITS2 using the RDP Naive Bayesian Classifier algorithm (Wang et al., 2007). ASVs assigned with no kingdom-level classification or defined as “Unknown” at the phylum rank were removed. ASVs of 16S data classified as “Chloroplast,” “Mitochondria” or “Eukaryota” and ASVs of ITS data classified as “Bacteria” or “Plant” were further removed. Sequence alignment was performed with AlignSeqs function from DECIPHER package (v.2.20.0). The phylogenetic tree was built using phangorn package (v.2.8.1) and then rooted for downstream reproducible analysis. The relative abundance tables for taxa were generated based on the read count for each taxon across samples using the total-sum scaling (TSS) method (Weiss et al., 2017). Diversity indices were calculated using phyloseq package (v.1.40.0) (McMurdie and Holmes, 2013) and visualized using ggplot2 package (v.3.3.5). Specifically, alpha diversity indices were calculated for each sample with not-rarefied data (McMurdie and Holmes, 2014). Beta diversity was performed using Principal Coordinates Analysis (PCoA) method with Unweighted distance in not-rarefied data, considering phylogenetic relatedness between samples in calculations.

### Comparison analysis of microbial communities across locations, compartments, and rootstock genotypes

Significant differences across locations were determined with one-way Analysis of Variance (ANOVA) followed by Tukey HSD *post hoc* test at *p*-value<0.05, prior checked the normal distribution of variable by Shapiro–Wilk test. Samples related to root and corresponding rhizosphere associated to different rootstocks were grouped according to the shared rootstocks (Supplementary Table S1). Specifically, six rootstocks which were present in at least three locations were selected: Swingle citrumelo [*Citrus paradisi* Macf. × *P. trifoliata* (L.) Raf.], Rangpur lime [*C. limonia* L. Osb.], Trifoliate orange [*C. trifoliata* (L.) Raf.], Ortanique tangor [*C. sinensis* L. Osb. × *C. reticulata* Bl.], Citrange [*C. sinensis* (L.) Osb. × *C. trifoliata* (L.) Raf.] ‘Carrizo’ and ‘Troyer’. Samples associated with other rootstocks with less than three replicates were discarded from further analyses. The significant differences in alpha diversity across compartments of same locations and rootstock genotypes were determined by one-way ANOVA and Fisher’s least significant difference (LSD) *post hoc* test at *p*-value<0.05. In addition, two-way ANOVA effect with interaction between the fixed variables compartment, location and rootstock genotypes was performed. Statistical analyses of beta diversity were performed by permutational multivariate analysis of variance (PERMANOVA), using a pairwise multilevel comparison throughout *Adonis* (Anderson, 2001) command in *vegan* package (v.2.5.7), with a permutation number of 999 available.

Based on the read count abundance profiles, the features (phylum, genus) with significantly differential abundances across compartments (root versus corresponding rhizosphere) were determined using *DESeq2* tool (v.1.34.0) (Love et al., 2014), fitting the negative binomial model and testing the significance with *Wald test*. Analyses of Compositions of Microbiome with Bias Correction (ANCOM-BC) among rootstocks for both ITS and 16S, in Root and Rhizosphere at Class level were performed using*ANCOMBC* package (Lin and Peddada, 2020). *p*-values for multiple tests were corrected using the BH method. All items with adjusted *p*-value<0.05 were considered significant. Moreover, based on the relative abundance of bacterial and fungi ASVs, the pairwise spearman’s correlation coefficient (ρ) values were calculated. Only correlations between two ASVs were considered as statistically robust if |ρ| ≥0.65 (Spearman’s rank correlation coefficient) and the *p*-value<0.05. The degree, betweenness, and modularity of each network were calculated using *igraph* package (v. 1.5.1). The co-occurrence networks were visualized using the *ggnet2* package (v. 0.1.0).

### Characterization and functional analysis of the core microbiome

Core members of the root and rhizosphere were calculated using the occurrence of association across samples as the criterion (Neu et al., 2021) using *Core* function in *microbiome* package (v.1.5.28) (Lahti and Shetty, 2017). Specifically, the genera that were present in more than 75% of samples across the globe for each compartment were considered as core genera (Xu et al., 2018). To generate the potential functions of core root microbiome, we first identified the genes that belonged to these core genera from our global citrus rhizosphere microbiome gene sets (Xu et al., 2018). Then according to the functional annotation of identified genes, the potential functions and KEGG pathways of core genera of root microbiome were inferred. The enrichment analysis of functional pathways compared to the functions of whole gene set were performed using fishers’ exact test based on *p*-value was less than 0.05.

## Results

### Overview of the global citrus root microbiota based on the 16S rDNA and ITS2 amplicon sequencing

**Figure 1:**
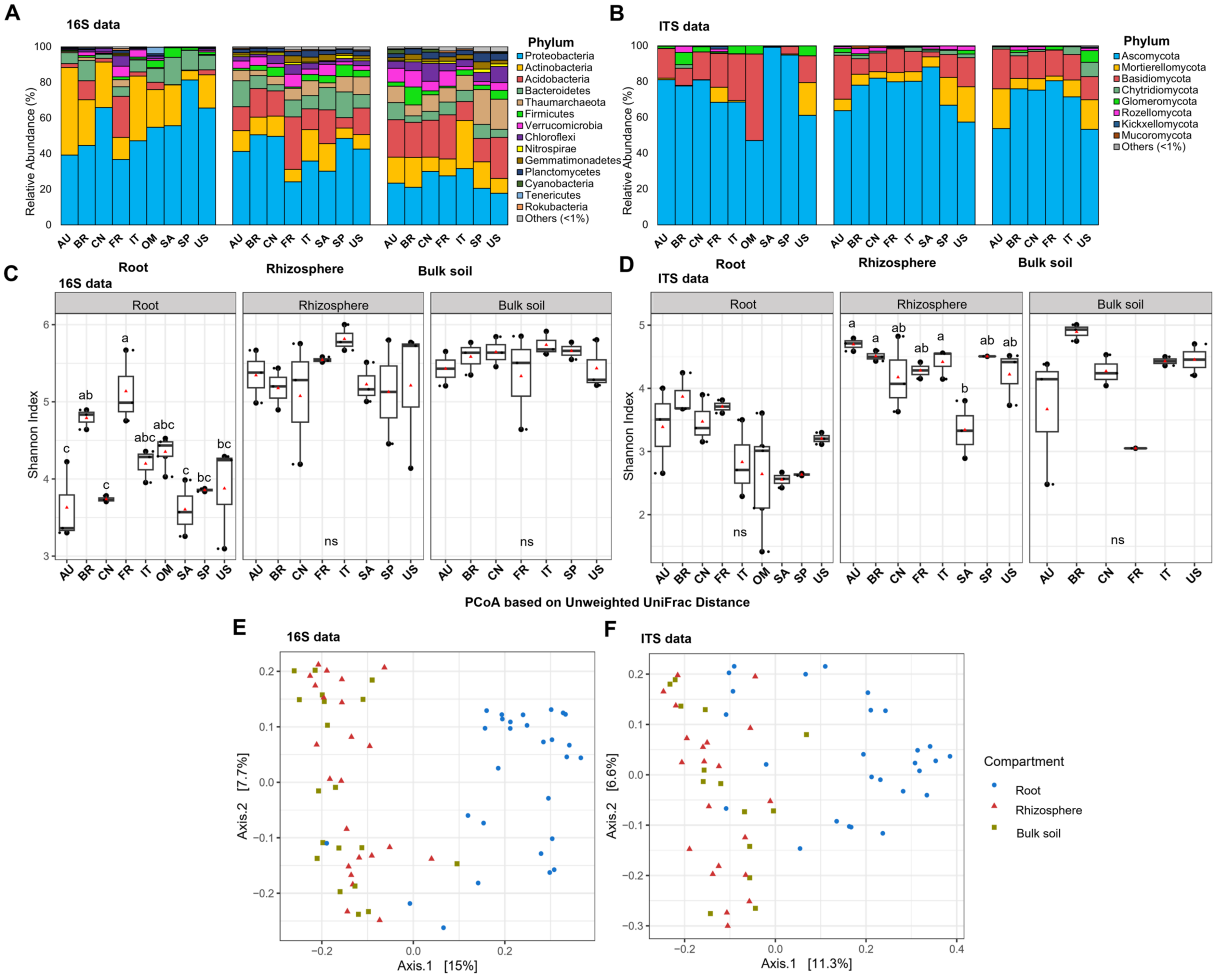
Taxonomic and diversity comparison across compartments and locations. **(A)** Relative abundance of prokaryotic phyla based on 16S amplicon data in samples from different locations in root and corresponding rhizosphere and bulk soil. **(B)** Relative abundance of fungal phyla based on ITS amplicon data in samples from different locations in root and corresponding rhizosphere and bulk soil. **(C)** Alpha diversity comparison between different locations in root and corresponding rhizosphere and bulk soil based on the Shannon index using the 16S amplicon data. **(D)** Alpha diversity comparison between different locations in root and corresponding rhizosphere and bulk soil based on the Shannon index using the ITS amplicon data. Different letters in the top of bars in panels **(C,D)** indicate significant difference among locations, *p*-value<0.05; ns = not significant at *p*-value<0.05; One-way ANOVA followed by Tukey’s multiple comparison test. The center value represents the median of alpha index. Points represent random variation on the location of each point. **(E)** PCoA based on the unweighted Unifrac distance across compartments using 16S data. **(F)** PCoA based on the unweighted Unifrac distance across compartments using ITS data. AU Australia, BR Brazil, CN China, FR French Réunion island, IT Italy, OM Oman, SA South Africa, SP Spain, US United States.

The clean dataset obtained from root samples comprised a total of 28 samples from nine countries spanning in all the six continents (Supplementary Table S1) where citrus grows for 16S rDNA amplicon analysis and 27 samples for ITS2 amplicon analysis. A sample with fewer than 1,000 high-quality reads was excluded from the ITS dataset. The dataset contained an average of 8,166 and 17,930 read counts per sample for 16S and ITS, respectively (Supplementary Tables S2, S3). A total of 2,586 distinct prokaryotic (bacterial and archaeal) ASVs encompassing 31 phyla and 2,812 distinct fungal ASVs encompassing 12 phyla were identified using DADA2 pipeline (Supplementary Table S4). We defined the prokaryotic and fungal phyla that exhibited relative abundance >1% in at least one location as the predominant ones (prokaryotic, Figure 1A; and fungal, Figure 1B). Ten predominant prokaryotic phyla, including 9 bacterial phyla and 1 archaeal phylum (Thaumarchaeota), were identified in the citrus root samples, with Proteobacteria, Actinobacteria, Acidobacteria, and Bacteroidetes being the top dominant phyla identified (Figure 1A). Regarding the fungal phyla, the representatives included Ascomycota, Basidiomycota and, to a lesser extent, Glomeromycota and Mortierellomycota, together accounting for more than 90% of the total phyla in each location (Figure 1B).

### Comparison of citrus root, rhizosphere and bulk soil microbiota

**Figure 2:**
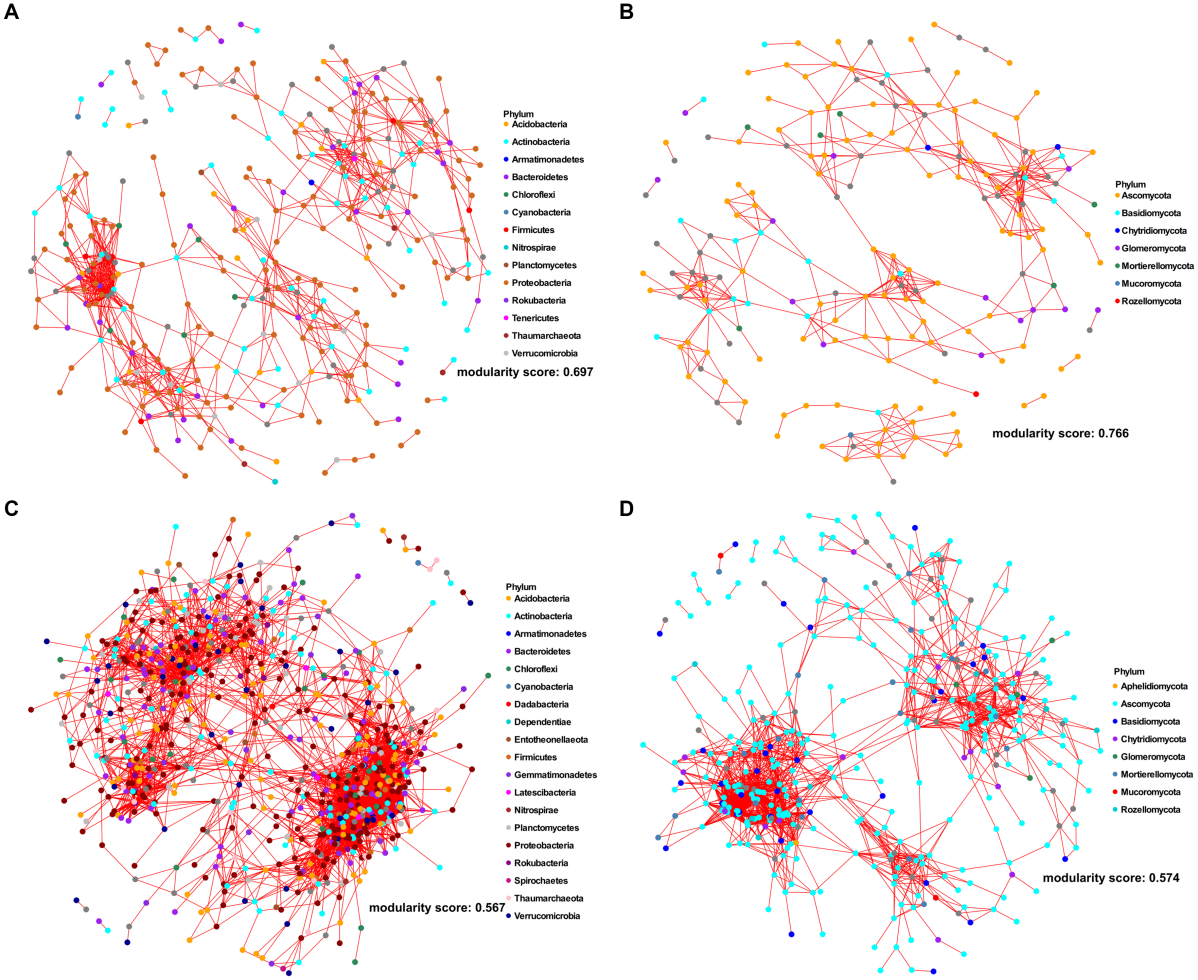
Co-occurrence networks of root **(A,B)** and rhizosphere **(C,D)** for bacterial **(A,C)** and fungal **(B,D)** ASVs, respectively. The networks were constructed based on Spearman correlation analysis of taxonomic profiles, with |ρ| ≥0.65 and the *p*-value <0.05. The nodes and the edges represent microbial ASVs and the correlations among them. Each node was colored according to the phylum taxonomic level.

To gain insights in the assembly clue of root microbiota, we analyzed the 16S rDNA and ITS2 amplicon sequencing data from the corresponding rhizosphere and bulk soil samples from the same trees (Xu et al., 2018) using the identical analysis pipeline as mentioned above. The alpha-diversity of both the root prokaryotic and fungal communities was lower compared to the adjacent rhizosphere samples. This was evident from the results obtained through both location-specific and overall comparative analyses (Figures 1C,D; Supplementary Figure S1, and Supplementary Tables S5, S6) (*p*-value <0.05; One-way ANOVA followed by Fisher’s least significant difference test). Beta-diversity analysis revealed that the root samples clearly clustered distinctly apart from the bulk soil and rhizosphere, forming two separate groups for both prokaryotic (Figure 1E, *R*^2^ = 0.05; *p* < 0.005, PERMANOVA) and fungal (Figure 1F, *R*^2^ = 0.09, *p* < 0.001, PERMANOVA) communities. Moreover, network analyses were performed to compare the inter-member interaction attributes between the root and rhizosphere microbial communities (Supplementary Datas S1–S4). The root prokaryotic network consisted of 334 nodes with an average degree number of 5.84 and modularity score of 0.697, which suggested that the root prokaryotic community formed a modular structure based on the modularity score threshold of 0.634, as described by Newman (2006); the corresponding rhizosphere co-network consisted of 781 nodes with an average degree number of 14.18 and modularity score of 0.567, indicative of a non-modular structure (Figures 2A,C). The root fungal network comprised of 197 nodes, with an average degree number of 4.72 and modularity score of 0.766, while the rhizosphere network comprised of 385 nodes, with an average degree number of 10.62 and modularity score of 0.574 (Figures 2B,D). These results suggested that the root prokaryotic and fungal communities were less diverse and complex but more modular than the rhizosphere ones.

In the rhizosphere and bulk soil microbiota, more predominant prokaryotic (12 phyla for rhizosphere and 13 phyla for the bulk soil) and fungal (five phyla for rhizosphere and six phyla for the bulk soil) phyla were identified than the root microbiota (Figures 1A,B). Further comparative microbiota analyses were conducted, and the results suggested that no bacterial phylum was enriched, and 20 phyla were depleted in the root microbiota compared with the corresponding rhizosphere (corrected *p*-value<0.05, DESeq2, Supplementary Table S7). One fungal phylum, Glomeromycota, was enriched in the root niche, while Kickxellomycota, Mortierellomycota, Chytridiomycota, Monoblepharomycota, Aphelidiomycota and Blastocladiomycota were depleted in the root microbiota compared with the rhizosphere microbiota (corrected *p*-value<0.05, DESeq2, Supplementary Table S7). At the genus level, 30 root-enriched and 150 root-depleted genera in bacterial community were identified (corrected *p*-value<0.05, DESeq2, Supplementary Data 5). For the fungal community, 21 genera were enriched, and 147 genera were depleted in the root niche compared with the rhizosphere (corrected *p*-value<0.05, DESeq2, Supplementary Data 5). Moreover, 62 bacterial genera and 61 fungal genera were specifically identified in the roots but not identified in the rhizosphere (Supplementary Table S8).

### Effect of citrus genotype on the microbial diversity of the root prokaryotic and fungal communities

**Figure 3:**
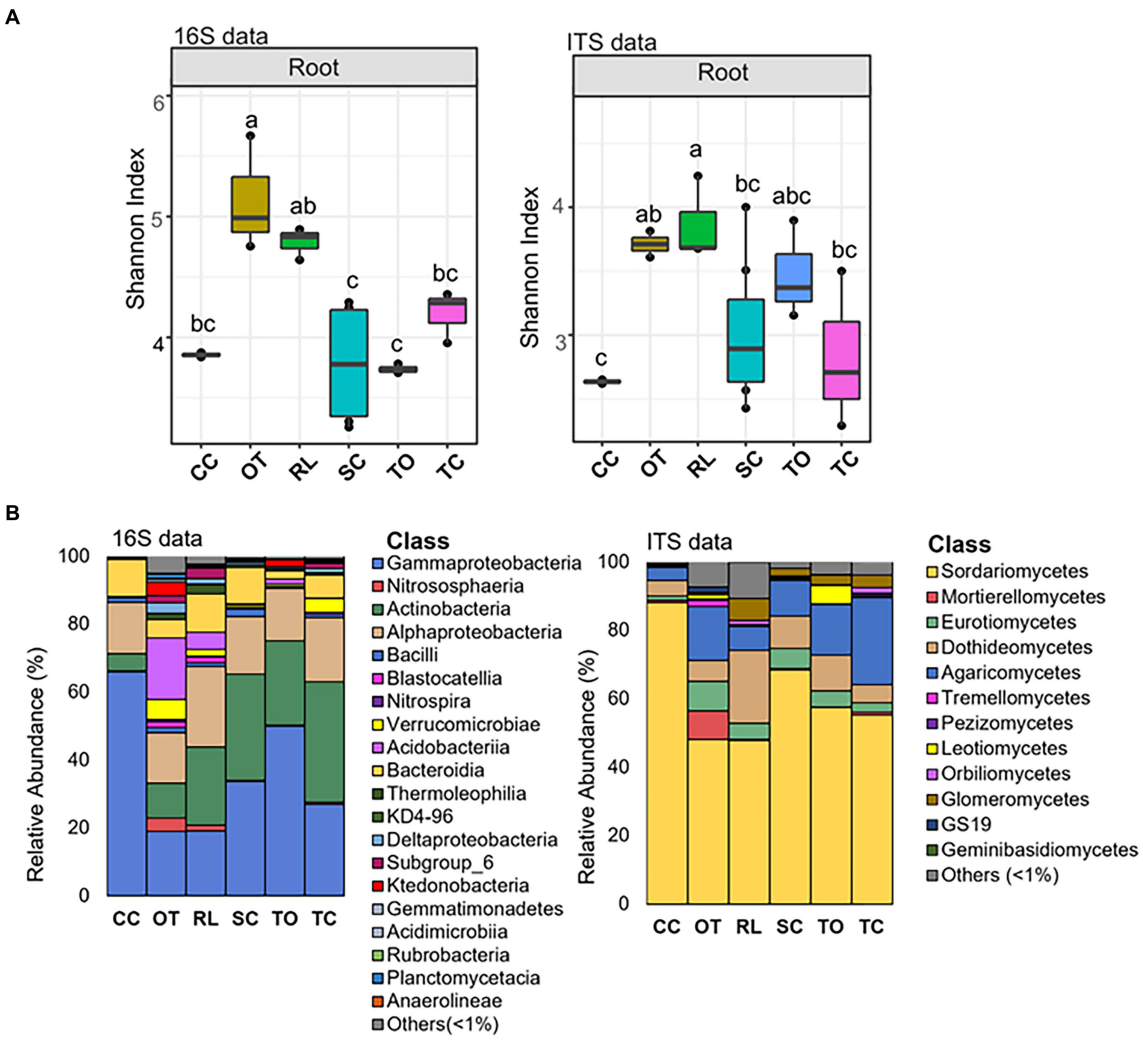
Alpha diversity and taxonomic comparison of root microbiota across rootstocks. **(A)** Alpha diversity comparison across rootstocks based on the Shannon index using the 16S (left) and ITS (right) amplicon data. Different letters in the top of bars indicate significant difference among rootstocks, *p*-value<0.05; One-way ANOVA followed by Fisher’s least significant difference (LSD) test. The center value represents the median of values. **(B)** Relative abundance of prokaryotic (left) and fungal (right) classes based on 16S and ITS amplicon data in samples from different rootstocks. CC, Carrizo citrange; OT, Ortanique tangor; RL, Rangpur lime; SC, Swingle citrumelo; TO, Trifoliate orange; TC, Troyer Citrange.

Interestingly, while the alpha-diversity of the prokaryotic community of citrus rhizosphere (*F*_7,22_ = 0.60; *p* = 0.746) and bulk soil (*F*_6,18_ = 0.56, *p* = 0.751) exhibited minimal variations across different geographic locations, the alpha-diversity of the root prokaryotic community displayed significant fluctuations across these geographic areas (*F*_8,27_ = 6.73, *p* < 0.05) (Figure 1C and Supplementary Table S5). For the fungal communities, no statistically significant differences were identified for the root and bulk soil samples across all locations (Figure 1D) (*F*_8,25_ = 2.32; *p* = 0.069, root; *F*_5,13_ = 2.53, *p* = 0.117, bulk soil); however, the rhizosphere fungal community exhibited significant variations in term of alpha-diversity (*F*_7,20_ = 3.80; *p* < 0.05) (Figure 1D and Supplementary Table S6). Because citrus genotype is one of the most significant variables in different locations, we further conducted citrus genotype-based group-wise comparisons. The results revealed that the citrus genotype indeed had strong influences on the root prokaryotic and fungal communities. The alpha diversity of root prokaryotic (*F*_5,21_ = 9.41, *p* < 0.05) and fungal (*F*_5,20_ = 3.05, *p* < 0.05) communities were significantly different among the six citrus genotypes (Figure 3A), while no significant difference was observed for the rhizosphere prokaryotic (*F*_5,21_ = 1.21, *p* > 0.05) and fungal (*F*_5,19_ = 0.903, *p* > 0.05) communities (Supplementary Figure S2A). Moreover, the beta-diversity comparison demonstrated that the factor “genotype” exerted a pronounced influence on the root prokaryotic (*R*^2^ = 0.36, *p*-value<0.001, PERMANOVA) and fungal community (*R*^2^ = 0.35, *p*-value<0.001, PERMANOVA).

Several predominant taxa exhibited dramatically varied relative abundance among different citrus genotypes (Figure 3B; Supplementary Figure S2B, and Supplementary Table S9). For instance, the relative abundance of class *Gammaproteobacteria* and *Bacteroidia*, two of the dominant classes in both root and rhizosphere prokaryotic community, were higher in Carrizo citrange than Trifoliate orange, while the relative abundance of class *Acidobacteriia* exhibited an opposite trend with relative abundance higher in Trifoliate orange than Carrizo citrange (Figure 3B; Supplementary Figure S2B, and Supplementary Table S9). Similarly, variations in the relative abundance of several taxa in the root and rhizosphere fungal community were also observed (Figure 3B; Supplementary Figure S2B, and Supplementary Table S9).

### Core members of global citrus root microbiota

**Figure 4:**
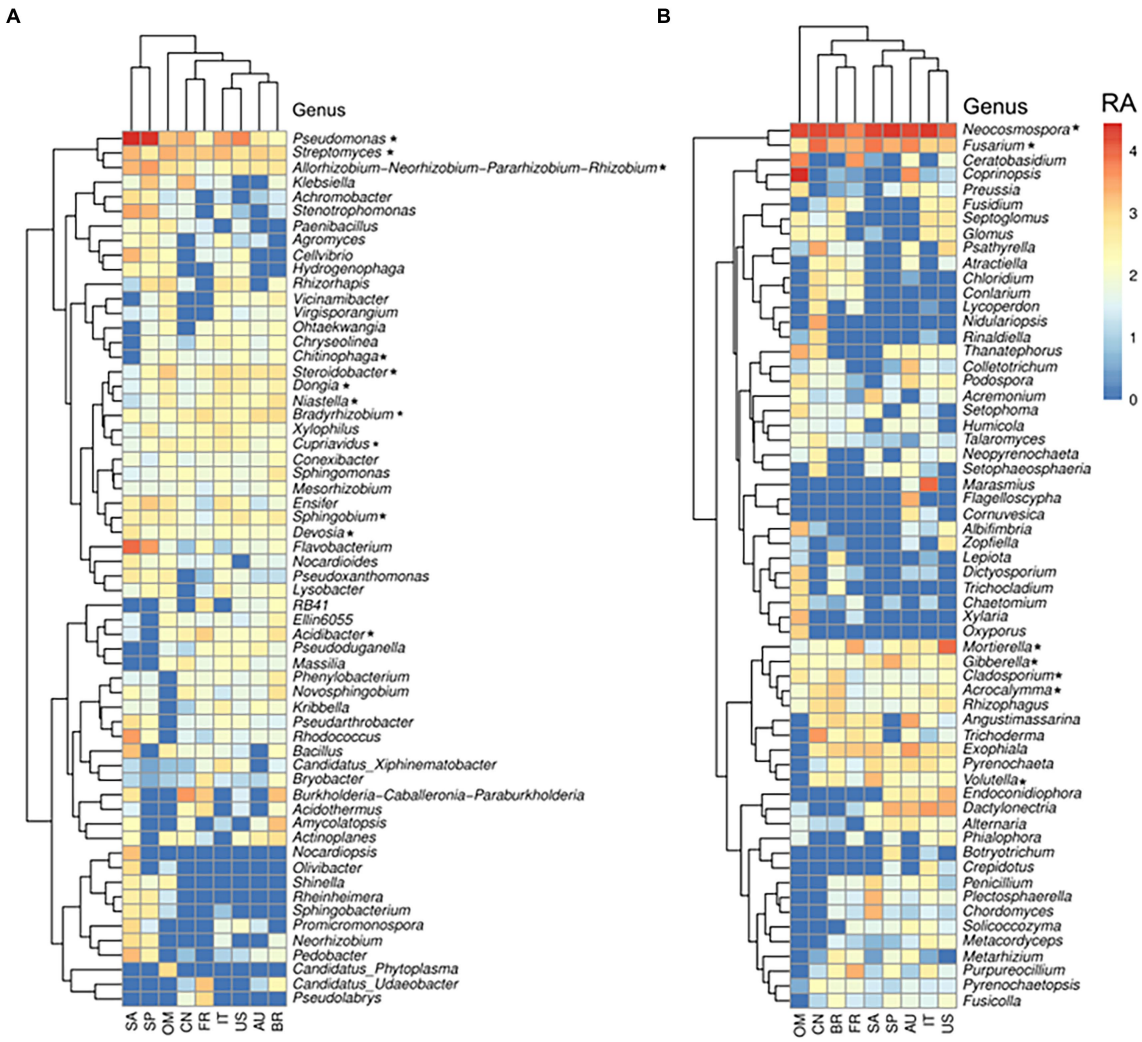
Relative abundance of the most abundant (top 60) bacterial **(A)** and fungal **(B)** genera in the root across locations. Genera signed with * were the core citrus root microbiome, based on the presence rate > 75% across all samples. The color from blue to red represents a relative abundance of each taxon from low to high. Scale, relative abundance (RA) of genus at row normalization of observed reads by Log10 (x + 1). AU Australia, BR Brazil, CN China, FR French Réunion island, IT Italy, OM Oman, SP Spain, US United States.

In this study, the taxa that were present in >75% of the root samples were defined as core taxa of citrus root microbiota, and 12 core prokaryotic genera and 7 core fungal genera were identified (Supplementary Data 6). The core prokaryotic genera included *Acidibacter*, *Allorhizobium*, *Bradyrhizobium*, *Chitinophaga*, *Cupriavidus*, *Devosia*, *Dongia*, *Niastella*, *Pseudomonas*, *Sphingobium*, *Steroidobacter*, and *Streptomyces*. All of these genera were among the most abundant genera in root samples (Figure 4A). Moreover, all the identified core genera, which contained several ASVs, were present within the co-occurrence network, and particularly, the ASVs members belonging to genus *Pseudomonas* were identified as having both high number of degree (number of connections of each node) and high values of betweenness centrality (importance for network connectivity), representing putative hubs and key connectors (Supplementary Data 1). Eleven of the 12 genera were also identified as the core members in the citrus rhizosphere microbiota (Supplementary Data 7), and *Allorhizobium* exhibited significantly higher relative abundance in the root samples than in the rhizosphere ones (corrected *p*-value<0.05, DESeq2; Supplementary Data 1). Of note, *Chitinophaga* was identified as a core genus in the root microbiota and not in the rhizosphere microbiota. Furthermore, *Chitinophaga* only exhibited relatively low relative abundance in 17 of 23 rhizosphere samples (Supplementary Datas 6, 7). The core fungal genera in the citrus root microbiota included *Acrocalymma*, *Cladosporium*, *Fusarium*, *Gibberella*, *Mortierella*, *Neocosmospora* and *Volutella* (Supplementary Data 6). These fungal genera were also among the most abundant fungal genera identified in the root compartment across globe (Figure 4B), and all identified taxa were present within the root co-occurrence networks and majority of them exhibited high scores of betweenness centrality (Supplementary Data 2). All fungal genera were also identified as core genera in the citrus rhizosphere microbiota. Among them, *Neocosmospora* exhibited significantly higher relative abundance in the root samples than in the rhizosphere ones (corrected *p*-value<0.05, DESeq2; Supplementary Data 5); and *Fusarium*, *Gibberella* and *Mortierella* exhibited relatively lower abundance in the roots (Supplementary Datas 6, 7).

**Figure 5:**
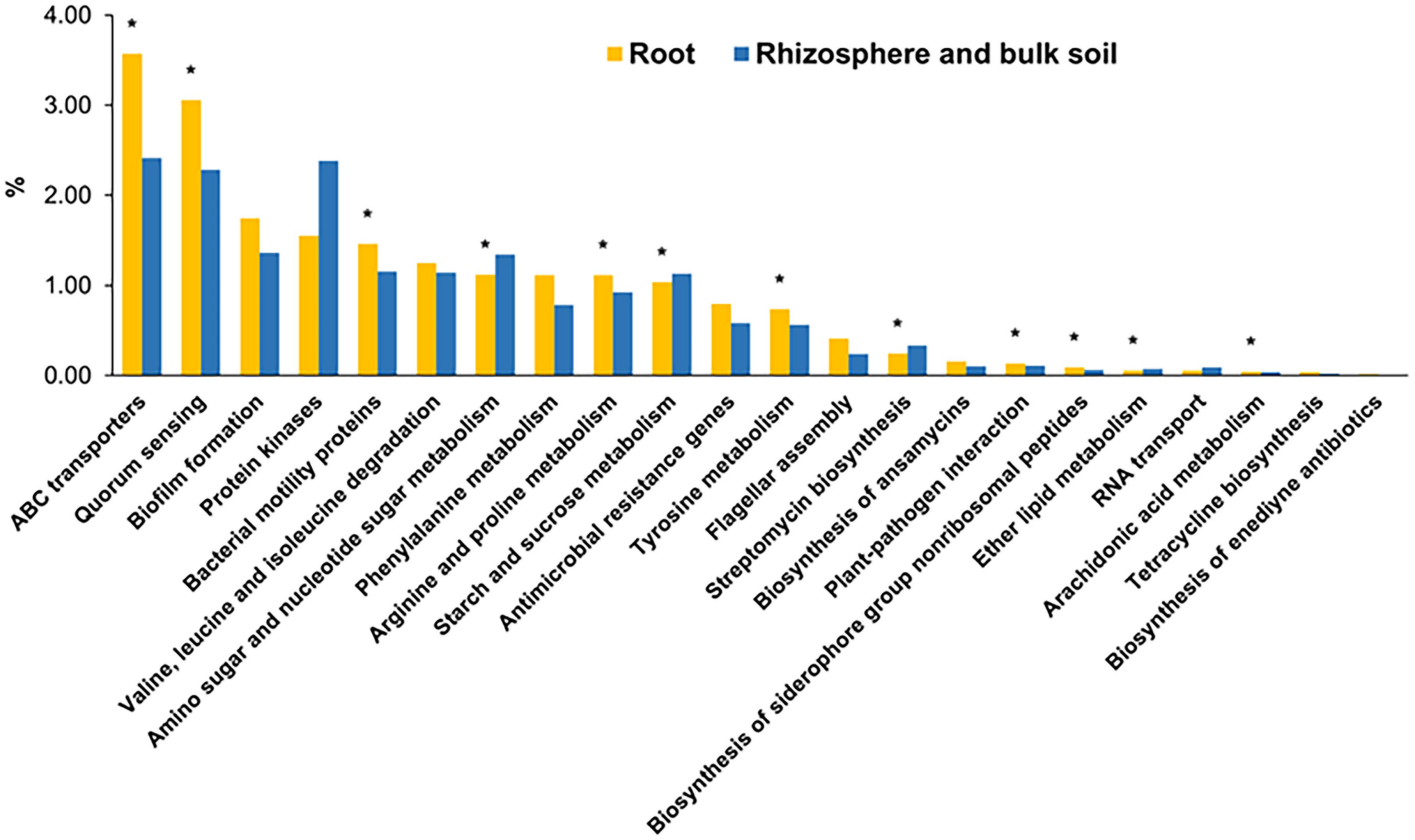
The overrepresented KEGG level 3 pathways for core root microbiome. The enrichment analysis of functional pathways compared to the functions of whole gene set were performed using fishers’ exact test based on *p*-value was less than 0.05. *corrected *p*-value <0.05.

Next, the potential functions of these core genera of root microbiota were predicted based on our previously established gene catalog of the global citrus rhizosphere microbiome (Xu et al., 2018). The predicted functions were distributed in 211 level 3 KEGG pathways (Supplementary Data 8). The functions of these root core genera were enriched in several KEGG pathways, including “ABC transporters,” “quorum sensing,” “biofilm formation,” “bacterial motility proteins,” “arginine and proline metabolism,” and “tyrosine metabolism,” compared with the rhizosphere (*p* < 0.05, fishers’ exact test) (Figure 5). On the other hand, the pathways “peptidases,” “amino sugar and nucleotide sugar metabolism,” and “starch and sucrose metabolism” were depleted in the root samples compared with the rhizosphere ones (*p* < 0.05, fishers’ exact test) (Figure 5).

## Discussion

In this study, the structure and diversity of global citrus root microbiota were investigated, and the prokaryotic members in the microbiota were predominantly populated by phyla Proteobacteria, Actinobacteria, and Bacteroidetes, while Ascomycota and Basidiomycota were the predominant fungal phyla in the root microbiota (Figure 1). The distribution of the predominant phyla in the citrus root microbiota was not only consistently with the neighboring rhizosphere microbiota (Xu et al., 2018) but also the root-associated microbiota from other plant species (Zhang et al., 2021). Majority of the core root microbiota were found to overlap with those of the rhizosphere (Figure 5 and Supplementary Data 3). These results suggested that the citrus root microbiota was largely recruited horizontally from the corresponding rhizosphere (Reinhold-Hurek et al., 2015; Liu et al., 2017; Deyett and Rolshausen, 2020; Wang et al., 2020). Of note, several taxa present in both citrus roots and rhizosphere exhibited significantly differential relative abundance and inter-microbe interactions between the two niches, demonstrating that the preference of these differentially abundant taxa and differences in the selective pressures between the two niches (Reinhold-Hurek et al., 2015; Wang et al., 2020). Several taxa such as *Chitinophaga* were identified as the core members in the citrus root microbiota but not in the rhizosphere microbiota, which suggested that the root microbiota may have other origins besides the adjacent rhizosphere, such as a vertical heritage from seed or internal migration from other plant organs (Ramírez-Puebla et al., 2013). Previous studies also demonstrated that the members in the root microbiota are assembled through horizontal transmission from the environment to host and vertical transmission from parent to offspring or via vectors (Reinhold-Hurek and Hurek, 2007; Hardoim et al., 2008; Marasco et al., 2018; Luo et al., 2019).

The host genotype has been demonstrated to play an important role in shaping its microbial component (Birt et al., 2022; Castellano-Hinojosa et al., 2023; Xu et al., 2023), resulting in varied distribution and abundance of microbes between different genotypes; however, certain microorganisms are found to be widely shared among diverse genotypes, thus maintaining common core members in plants phylogenetically close (Lemanceau et al., 2017). Here, we identified the core microbiota and their functions within the citrus root microbiota shared among different rootstock genotypes and growth conditions. Our analysis revealed that the bacteria belonging to the genera *Acidibacter*, *Allorhizobium*, *Bradyrhizobium*, *Cupriavidus*, *Chitinophaga*, *Devosia*, *Dongia*, *Niastella*, *Pseudomonas*, *Sphingobium*, *Steroidobacter*, and *Streptomyces* are core members of the citrus root microbiota. The core fungal root microbiota was significantly less diverse than the bacterial counterpart (Zhang et al., 2021) and includes the genera *Acrocalymma*, *Cladosporium*, *Fusarium*, *Gibberella*, *Mortierella*, *Neocosmospora*, and *Volutella*. Identification of beneficial microbes from the plant microbiota and application of them to improve plant health has shown great promise, including in citrus (Trivedi et al., 2010; Riera et al., 2017; Blacutt et al., 2020; Poveda et al., 2021). The development of bioinoculant from to the core members of citrus have been suggested (Zhang et al., 2021), and preliminary results suggested that the core microbiota associated with citrus leaves and roots has shown promising effects on the management of HLB disease (Blaustein et al., 2017). Among the identified core genera in the citrus root microbiota, the predominantly abundant genus *Pseudomonas* was well-established as a plant endophyte and extensively documented for its beneficial effects on plants as a core member, recently evidenced by Tian et al. (2017). It has been thoroughly studied for the biological control of citrus pathogens (De Oliveira et al., 2011; Trivedi et al., 2011; Liu et al., 2017; Poveda et al., 2021), making this core genus one of the most promising microbial members for use as bioinoculants (Singh et al., 2020; Zboralski and Filion, 2023). As another example, the successful application of a reconstructed synthetic endospheric consortium based on *Chitinopaga* genus provided a compelling demonstration of how a well-established root endophyte can be effectively employed as a bioinoculant (Carrión et al., 2019). The consortium effectiveness in suppressing fungal root diseases was attributed to the activation of enzymatic activities responsible for fungal cell-wall degradation, as well as the production of antifungal effectors and secondary metabolites. In this scenario, understanding the metabolic and functional processes associated with the bacterial core genera is critical for deciphering and explain their roles in interacting with the plant host and pathogens. Our results revealed an over-representation of the two-component system, ABC transporters and quorum sensing pathways in the root compartment, with the latter two showing significant enrichment. ABC transporters play a crucial role in the endosphere of plants, where they are involved in nutrient uptake, plant growth promotion, and stress tolerance (Santoyo et al., 2016; Do et al., 2018). Quorum sensing plays a major role in controlling various microbial cell activities, facilitating the movement and multiplication of endophytes inside the plant host, stimulating plant growth and eliciting defense responses (Mäe et al., 2001; Kumar, 2020; Paul et al., 2023). The presence of two-component systems in the bacterial core microbiota can enhance plant health by sensing environmental changes, transmitting the signals and then activating cellular responses (Stock et al., 2000; Groisman, 2016). This could help the plant respond more effectively to environmental stressors, nutrient acquisition, pathogen resistance, and promote overall growth and development (Borland et al., 2015). In addition, functions related to biofilm formation are also overrepresented. Biofilm formation is known to protect prokaryotes from environmental stress (Penesyan et al., 2021). Profiling the fungal core microbiota also revealed potential biocontrol agents for citrus main diseases, such as HLB (Passera et al., 2018). For example, *Cladosporium. cladosporioides* was shown to inhibit *Liberibacter crescens*, the culturable surrogate of *Candidatus* Liberibacter (CLas) (Blacutt et al., 2020). It was hypothesized that its dual nature, combining antagonism and endophytism, might lead to a colonization of the phloem, bringing it into direct contact with the pathogen.

These observations substantiate the notion that the development of efficient microbial consortium should start with the selection of highly specialized and stable core microorganisms (Choudhary and Schmidt-Dannert, 2010). The presence of these microorganisms in a global dataset further reinforces the viability of their widespread application, that could ensure the successful colonization of citrus plants following the field releases, which is as an important step required for exhibiting beneficial effects and overall efficacy beyond controlled laboratory conditions (Kong et al., 2018). Nonetheless, further isolation and *in vivo* application of these core members could unveil their effective potential as agricultural probiotics, offering promising prospects for future biotechnological applications within the citrus industry.

## Conclusion

This study provides new information about assemblage of microbial communities and core members of citrus root microbiota in a broad biogeographical sampling. Considering the assumption that the roots probably host a highly specialized and niche-adapted community, profiling of the core root microbial communities allows to identify target organisms that are strictly selected and adapted to their host. This knowledge may fuel the development of novel bioproducts with large-scale applications or help with the implementation of cultural practices associated with the selection of host genotype that support the presence of key beneficial microbes.

## Data availability statement

The raw sequencing reads were deposited in the NCBI database under the accession number BioProject PRJNA844917. The raw sequencing reads of the corresponding rhizosphere and bulk soil samples were deposited in the NCBI database under the accession number BioProject PRJNA362455.

## Author contributions

ML: Investigation, Writing – original draft, Writing – review & editing, Data curation, Formal analysis. YZ: Formal analysis, Investigation, Writing – original draft, Writing – review & editing, Methodology. JX: Formal analysis, Investigation, Methodology, Writing – original draft, Writing – review & editing. PT: Investigation, Methodology, Writing – review & editing, Resources. PZ: Investigation, Methodology, Formal analysis, Writing – review & editing. NR: Investigation, Methodology, Writing – review & editing, Formal analysis. LL: Investigation, Methodology, Resources, Writing – review & editing. YW: Investigation, Methodology, Resources, Formal analysis, Writing – review & editing. XL: Formal analysis, Investigation, Methodology, Writing – review & editing. GF: Formal analysis, Investigation, Methodology, Writing – review & editing. JT: Investigation, Methodology, Resources, Writing – review & editing. HC-F: Investigation, Methodology, Resources, Writing – review & editing. JC: Investigation, Methodology, Resources, Writing – review & editing. XD: Investigation, Methodology, Resources, Writing – review & editing. VA: Investigation, Methodology, Resources, Writing – review & editing. ZL: Investigation, Methodology, Resources, Writing – review & editing. BZ: Investigation, Methodology, Resources, Writing – review & editing. MR: Investigation, Methodology, Resources, Writing – review & editing. NC: Investigation, Methodology, Resources, Writing – review & editing. VC: Investigation, Methodology, Resources, Writing – review & editing. GP: Investigation, Methodology, Resources, Writing – review & editing. AA-S: Investigation, Methodology, Resources, Writing – review & editing. XX: Investigation, Methodology, Writing – review & editing, Formal analysis. JW: Formal analysis, Investigation, Methodology, Writing – review & editing. HY: Formal analysis, Investigation, Methodology, Writing – review & editing. TJ: Formal analysis, Investigation, Methodology, Writing – review & editing. GC: Formal analysis, Investigation, Methodology, Writing – review & editing. NW: Investigation, Writing – review & editing, Conceptualization, Funding acquisition, Project administration, Supervision, Writing – original draft.
