# Supplementary material for: Global citrus root microbiota unravels assembly cues and core members
Source: Front Microbiol. 2024 Jul 26;15:1405751. doi: 10.3389/fmicb.2024.1405751 (PMC11310164; doi:10.3389/fmicb.2024.1405751)
Supplement: Supplementary file 3 [file Image_1.pdf]

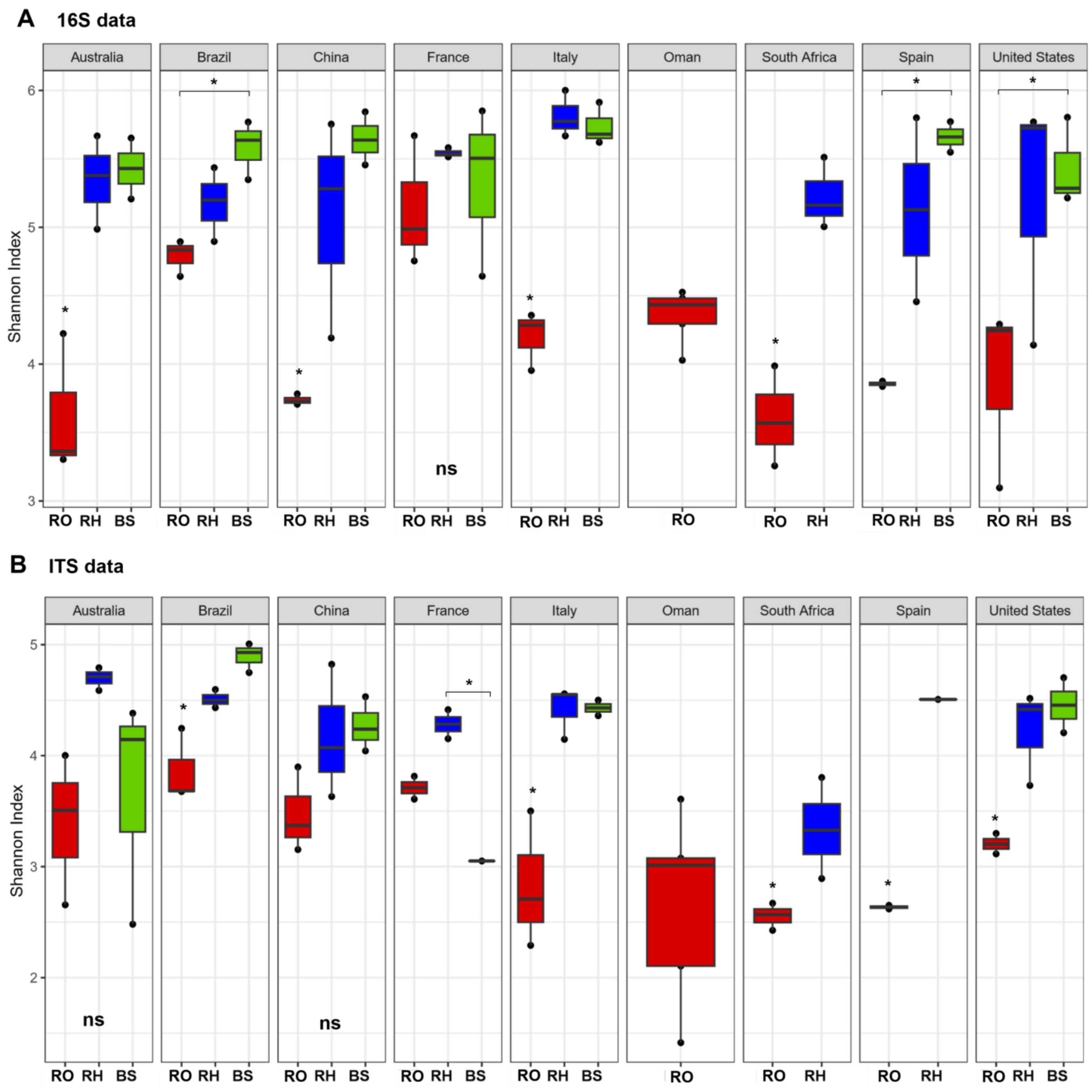

Fig. S1. Alpha diversity comparison between root and corresponding rhizosphere and bulk soil within each location based on Shannon index using 16S (A) and ITS (B) amplicon data; *, indicate significant difference within location, P-value<0.05; ns= not significant; One-way ANOVA followed by Fisher's least significant difference (LSD) test. RO, root (red), RH, rhizosphere (green), BS, bulk soil (blue). AU, Australia; BR, Brazil; CN, China; FR, French Réunion island; IT, Italy; OM, Oman; SA, South Africa; SP, Spain; US United States.

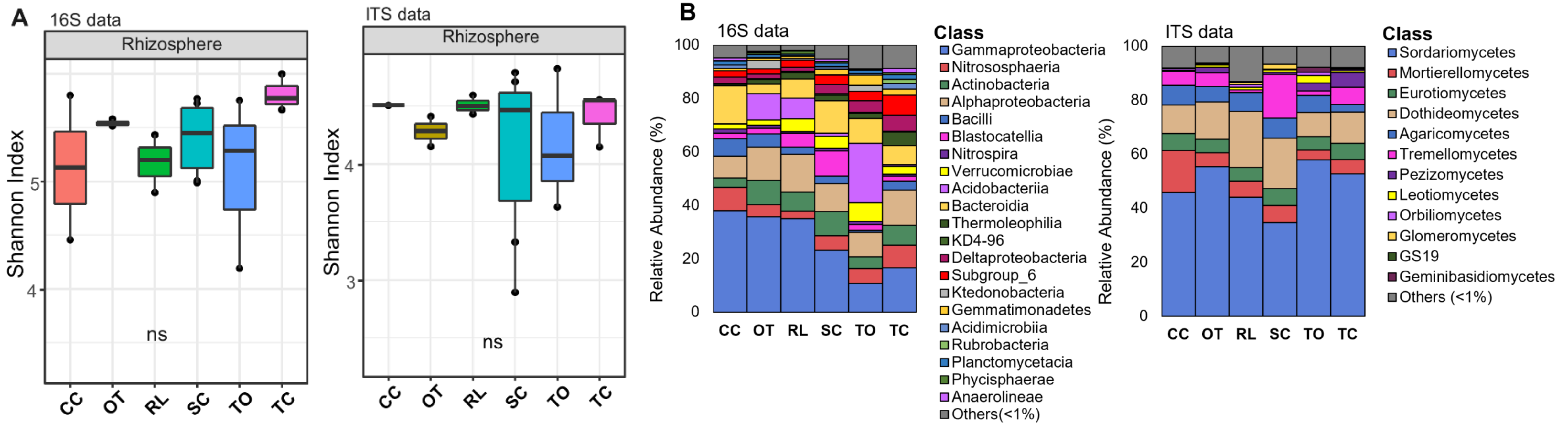

Fig. S2. Alpha diversity and taxonomic comparison of rhizosphere microbiota across rootstocks. A, alpha diversity comparison across rootstocks based on the Shannon index using the 16S (left) and ITS (right) amplicon data. Different letters in the top of bars indicate significant difference among rootstocks, P-value<0.05; One-way ANOVA followed by Fisher's least significant difference (LSD) test. The center value represents the median of values. B, relative abundance of prokaryotic (left) and fungal (right) classes based on 16S and ITS amplicon data in samples from different rootstocks. CC, Carrizo citrange; OT, Ortanique tangor; RL, Rangpur lime; SC, Swingle citrumelo; TO, Trifoliate orange; TC, Troyer Citrange.
